# Palms are unique: clade‐level pattern of the leaf-height-seed strategy scheme

**DOI:** 10.3389/fpls.2024.1465935

**Published:** 2024-11-01

**Authors:** Xiaolan Li, Shijia Fu, Mingming Zhang, Fei Yu, Yang Wang, Xianfeng Yi

**Affiliations:** ^1^ School of Resources and Environment, Yili Normal University, Yili, China; ^2^ College of Life Sciences, Henan Normal University, Xinxiang, China; ^3^ College of Agriculture, Henan University of Science and Technology, Luoyang, China; ^4^ Henan Dabieshan National Field Observation and Research Station of Forest Ecosystem, Zhengzhou, China; ^5^ School of Life Sciences, Qufu Normal University, Qufu, China

**Keywords:** LHS scheme, palms, other monocots, dicots, gymnosperms, phylogeny

## Abstract

**Introduction:**

The leaf-height-seed (LHS) plant ecology strategy scheme posits that functional traits such as leaf size, stem height and seed mass play a key role in life history of plants. Although many studies have explored the LHS scheme across plant species, to our knowledge, no study has so far linked functional trait patterns across different plant clades.

**Methods:**

Here, we first explored the LHS scheme of several plant clades, i.e., palms, other monocots, dicots and gymnosperms, to understand how potential forces drive variation of plant functional traits.

**Results:**

We showed that phylogeny constrains plant functional traits and appears to be the most decisive factor that controls variation in seed mass irrespective of plant clades. Apart from phylogeny, a majority of variation in seed mass was explained by leaf size in palms clade, whereas by plant height in other monocots and dicots. Neither leaf size nor plant height well explained variation in seed mass of gymnosperms clade.

**Conclusion:**

Our study strongly suggests that different plant clades exhibit distinct LHS schemes, paving a new avenue for better understanding evolution and correlation between functional traits across sets of plant species.

## Introduction

Plant functional traits (i.e., plant height, leaf area and seed size) have been considered as potentially powerful indicators of the ecological processes of species, which can also be used as indicators or reference for the maximum information of plant growth and resource utilization strategies (Wilson et al., 1999; Kooyma et al., 2010; Navarro et al., 2010; Adler et al., 2013). Plant traits have become a core attribute to determine plant strategies and then to understand and predict the evolution, distribution as well as ecological strategies of plant species at the scale of population, community and ecosystem (Gaudet and Keddy, 1988; Chapin et al., 2000; Kunstler et al., 2015), because they directly affect the basic behavior and function of plants, and reflect the survival strategies formed by plants adapting to environmental changes (Ackerly and Cornwell, 2007). Plant strategies can be quantified by measuring various functional characteristics that affect plant fitness and ecological processes (Lavorel and Garnier, 2002). Westoby chose the plant ecological strategy scheme (LHS), i.e., the use of three functional traits (Westoby, 1998), specific leaf area (SLA), plant canopy height and seed mass as representing three fundamental and relatively independent axes of a plant’s ecological strategy to classify plants according to meaningful axes of plant specialization (Vendramini et al., 2002; Laughlin et al., 2010; Díaz et al., 2015). Later on, ecologists have carried out a number of studies on the relationship between plant height, leaf area and seed mass (Lavergne et al., 2003; Wang et al., 2014; Wolf et al., 2022), and found consistent relationship between plant traits, which further improves our understanding of plant adaptation strategies (Koch et al., 2004).

Plant traits represent an outcome of evolutionary processes, therefore its distribution reliably reflects their evolutionary history and phylogenetic constrains (Wang et al., 2008; Reinhart et al., 2012). Phylogenetically related species share a common evolutionary history and may therefore have similar traits (Ibanez et al., 2016). Although the whole point of the scheme is that the LHS variables are not necessarily correlated with each other, much of the literature has provided evidence that the correlated evolutionary divergence of traits has led to trait correlations across plant species (Gingerich, 1974; Revell et al., 2008; Xia et al., 2022), such as correlation between leaf area and seed mass (Laughlin et al., 2010). McCarthy et al. (2007) and Reich et al. (2014) have shown that woody gymnosperms invest relatively more in leaves than woody angiosperms. Poorter et al. (2012) provided further evidence that herbaceous monocots have lower leaf mass fractions than herbaceous eudicots because dicots invest relatively more than monocots in leaves. Moreover, Damour et al. (2016) showed that dicots had higher seed mass than monocots. Differing from the other monocots (Raven, 1988; Tomlinson, 2006), palms build their tall primary stature and exhibit unique features such as leaf development and anatomical characteristics, and possibly the correlation of seed mass with leaf area and plant height (Moore, 2003; Sampaio and Scariot, 2008; Cámara-Leret et al., 2017). Therefore, it remains debatable if plant species from different clades will follow a specific LHS scheme at a higher classification level (e.g., genus), though variety of ecological strategy schemes have been proposed across plant species.

An important goal of plant ecology is to separate the key dimensions of ecological variations across species and then to understand how and why they function and vary between species. For example, the widely used LHS scheme of Westoby propose that each dimension of LHS vary widely between species at any given level of the other two, but it is not sufficient to describe the main axes of trait variation of temperate woody species (Westoby, 1998). Therefore, investigating the correlation of trait characters in different plant clades will provide a sound basis further our understanding of the evolution of functional characters among plants (Reich et al., 1999; Tjoelker et al., 2005; Pierce et al., 2014). In this context, “plant clades” refer to groups of plants that share a common evolutionary ancestor, allowing us to explore evolutionary relationships and trait variations across broad plant lineages. By analyzing these clades, we can better understand how evolutionary history influences ecological strategies and functional traits. However, to our knowledge, no study has so far investigated plant trait variation across different clades, especially using large datasets in the context of LHS scheme and phylogeny.

Consequently, it is still highly uncertain whether traits of different plant clades will fit a specific LHS scheme. Or, do all plant species within a specific clade support a plant ecological strategy scheme (LHS)? Although several authors have investigated LHS scheme within each clade such as palms, angiosperms, gymnosperms, annuals, perennials, herbaceous or woody plants (Falster and Westoby, 2005; Laughlin et al., 2010; Cámara-Leret et al., 2017; Kawai and Okada, 2020), to our knowledge no study has so far used large datasets to investigate correlations of plant traits across different plant clades (i.e., palms, other monocots, dicots, and gymnosperms). We addressed these questions by conducting a meta-analysis of functional traits (plant height, leaf size, and seed mass) from four plant clades, i.e., palms, other monocots, dicots and gymnosperms with contrasting growth forms. The primary aim of the current study was to understand if and how the different plant clades are coordinated along the plant ecological strategy scheme. Specifically, we first used phylogenetic generalized linear mixed models (PGLMM) and partial R^2^lik logistic regression model, to explore how potential forces drive variation in plant traits between different clades, so as to better understand evolution and correlation between functional traits among sets of plant species (Zheng et al., 2009). We expected that each clade of plant species will share the same plant ecological strategy scheme, while LSH scheme would differ across different plant clades.

## Materials and methods

### Data collection

Using Westoby’s leaf-height-seed (LHS) model of plant functional types (Westoby, 1998), we clustered the plant species studied into four clades: palms, other monocots, dicots, and gymnosperms. We extracted trait data of 2558 palm species, 160 genera from a species-level functional trait database of palms, Palm Traits 1.0 (Kissling et al., 2019), complemented with data from published literature (Göldel et al., 2015). Here, we focused on leaf size (maximum blade length in mm), stem height (maximum height in m), and fruit size (maximum fruit width in mm) to represent these major trait axes for palms. We used blade length as a proxy of leaf size of palms because it is commonly used in analyses of leaf traits (Göldel et al., 2015). Fruit size was used as a proxy for seed size because 1) little information of seed size is available for palms, 2) many palm genera are mainly 1-seeded, 3) fruit and seed size are often positively correlated (**Supplementary Figure S1**). Therefore, palms traits we collected are in line with the traits of the LHS plant ecology strategy scheme.

For all species of other monocots, dicots, and gymnosperms, we derived data of leaf size (in mm^2^), maximum plant height (in m) and seed mass (in mg) from TRY plant trait database and BEIN dataset (Fraser, 2020), complemented it with data from recent publication, representing the leaf–height–seed plant strategy scheme of Westoby (1998). In total, 836 species, 279 genera of other monocots, 4290 species, 1602 genera of dicots, and 112 species, 40 genera of gymnosperms were collected. All data of plant species were averaged at genus level before analysis, which will reduce the effect of environmental scales on plant functional traits.

### Statistical analysis

Pagel’s lambda (λ) is a robust estimate of the strength of phylogenetic signal in a continuous trait (Pagel, 1999; Münkemüller et al., 2012; Molina-Venegas and Rodríguez, 2017). In our study, Pagel’s λ can range from 0 to 1, a λ of 0 indicates that there is no phylogenetic signal in the focal traits, whereas a λ of 1 indicates high phylogenetic signal in which the focal trait evolved according to Brownian motion. We calculated Pagel’s to quantify the strength of the phylogenetic signal in plant traits (plant height, leaf size, seed mass, fruit width, blade length and stem height) of palms, other monocots, dicots and gymnosperms (Cadotte et al., 2013). We evaluated the importance of through randomized tests implemented in the function phylosig of the R package ‘phytools’ (Revell, 2011).

We used a Gaussian distribution with phylogenetic trees, implemented in the R packages ‘phyr’ and ‘ape’. The multivariate phylogenetic generalized linear mixed models (PGLMM) were used to test the effects of leaf size and plant height on seed mass while controlling for phylogeny.

The location of palms, other monocots, dicots, and gymnosperms in a multivariate trait space illustrated by the first two axes of the PCA based on traits of seed mass (fruit width), leaf size (blade length), and plant height (stem height) (Duras, 2020).

The partial R^2^ for the logistic regression model (Ives and Helmus, 2011) implemented by the R package “rr2” was used to tease apart the relative contributions of leaf size, plant height and phylogeny to the variation in seed mass of palms, other monocots, dicots, and gymnosperms. The partial R^2^lik for each factor was calculated by comparing the full model with reduced models in which a given factor was removed, and measuring the consequent reduction in the likelihood.

## Results

**Figure 1:**
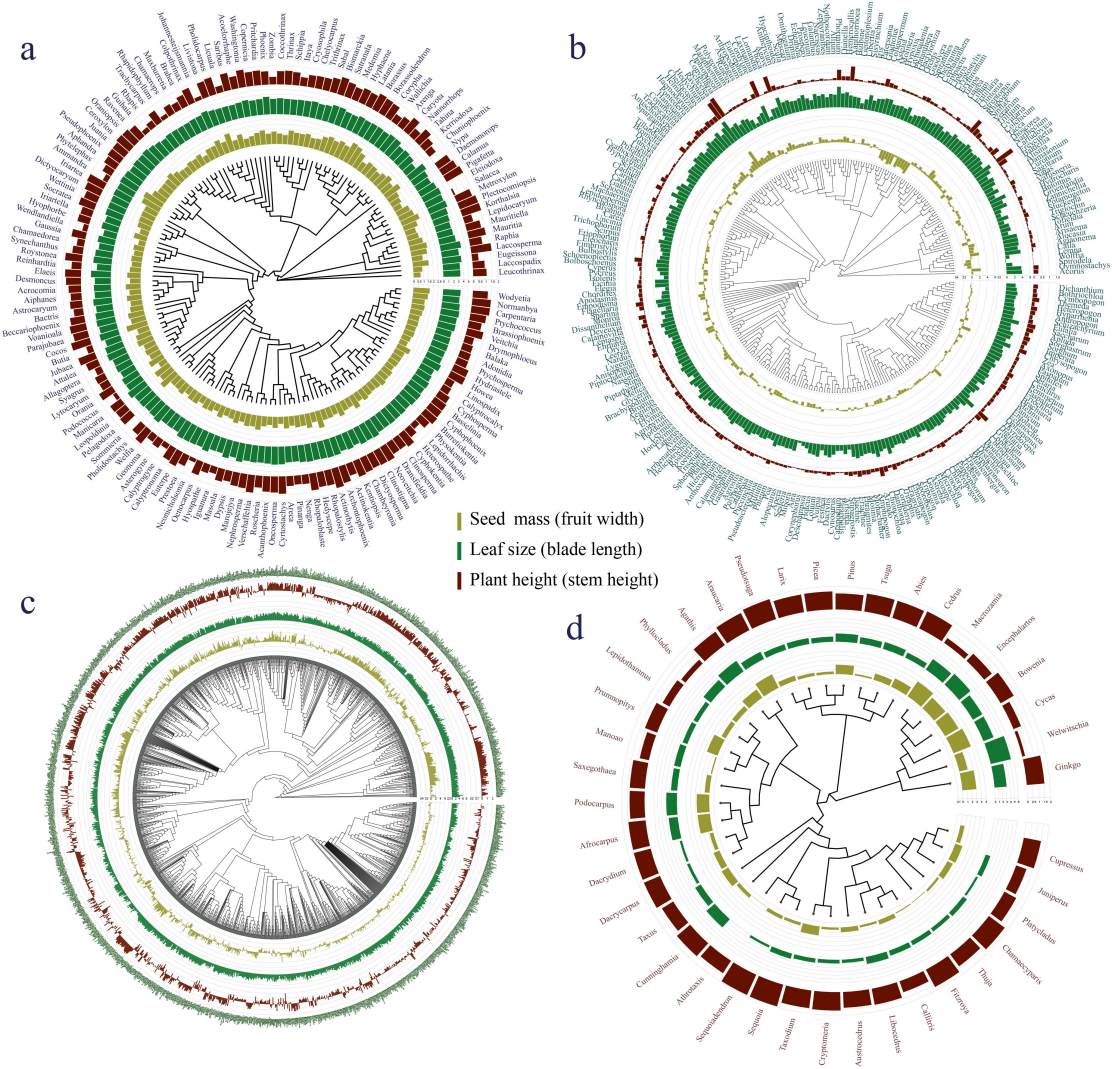
Plant key traits (plant height, leaf size, seed mass, fruit width, blade length and stem height) of **(A)** palms, **(B)** other monocots, **(C)** dicots, and **(D)** gymnosperms mapped onto a plant phylogeny. Bars at the phylogenetic tree indicate seed mass or fruit width (olivine), leaf size or maximum blade length (green) and maximum plant or stem height (dark brown).

**Figure 2:**
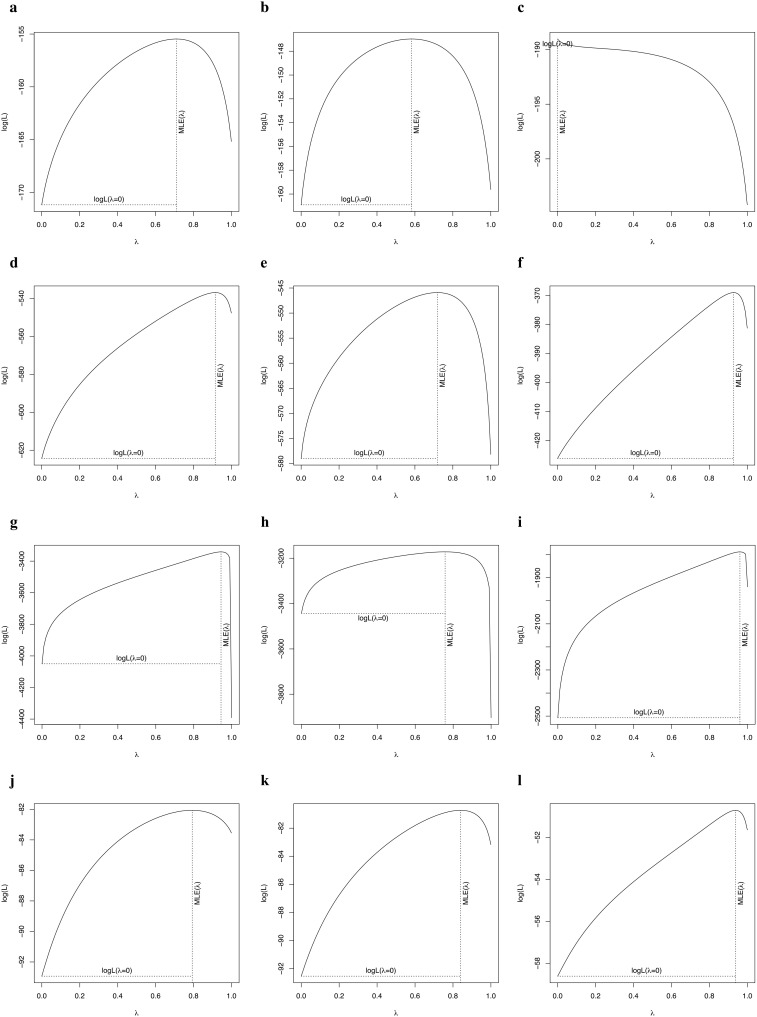
Phylogenetic signal test of key traits of palms (**A**: fruit width; **B**: blade length; **C**: stem height), other monocots (**D**: seed mass; **E**: leaf size; **F**: plant height), dicots (**G**: seed mass; **H**: leaf area; **I**: plant height), and gymnosperms (**J**: seed mass; **K**: leaf area; **L**: plant height).

The phylogenetic signals in seed mass (**Figures 1**, **2A, D, G, J**) and leaf size (**Figures 1**, **2B, E, H, K**) were moderate and statistically significant across palms, other monocots, dicots, and gymnosperms (**Table 1**), indicating that seed mass and leaf size have a common evolutionary history with species. We detected a strong phylogenetic signal in plant height of other monocots (**Figures 1B**, **2F**), dicots (**Figures 1C**, **2I**) and gymnosperms (**Figure 1D**, **2L**) but not palms (**Figures 1A**, **2C**; **Table 1**).

**Table 1:** Phylogenetic signal of functional traits of palms, other monocots, dicots, and gymnosperms as measured by Pagel’s λ (Pagel, 1999).

Taxon	Lambda value (*P*)		
	Seed mass (fruit width)	Leaf size (blade length)	Plant (stem) height
Palms	0.711 (< 0.001)	0.582 (< 0.001)	0.000 (1.000)
Other monocots	0.915 (< 0.001)	0.718 (< 0.001)	0.928 (< 0.001)
Dicots	0.944 (< 0.001)	0.757 (< 0.001)	0.960 (< 0.001)
Gymnosperms	0.793 (< 0.001)	0.840 (< 0.001)	0.938 (< 0.001)

**Figure 3:**
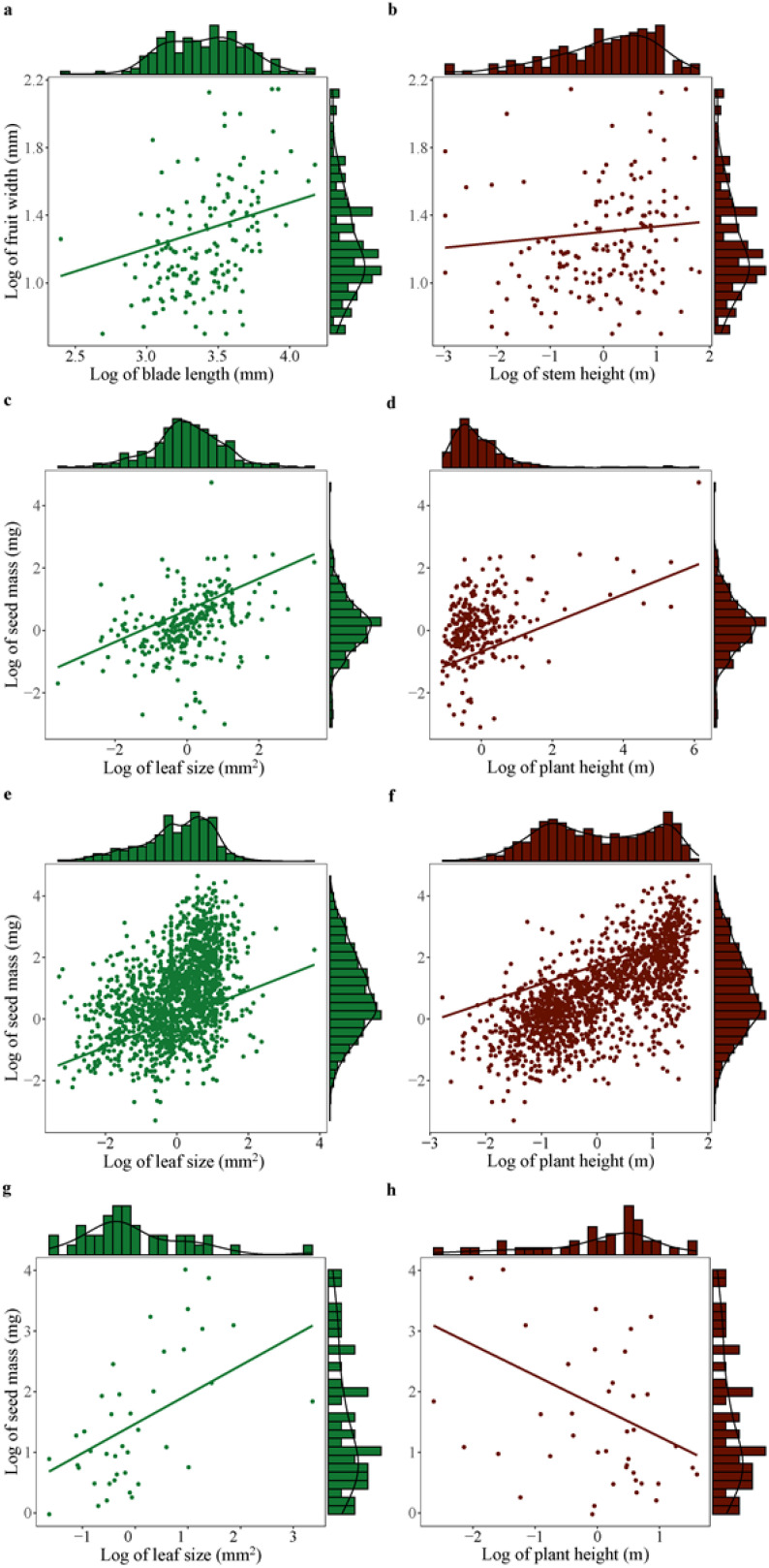
Significant correlation of seed mass (or fruit width) with leaf size (or blade length) and plant height (or stem height) across palms **(A, B)**, other monocots **(C, D)**, dicots **(E, F)**, and gymnosperms **(G, H)**. Significant effect was detected based on phylogenetic generalized linear mixed models (PGLMM, see **Table 2**).

When controlling for phylogeny, seed mass showed significantly positive correlation with leaf size across plant species (**Table 2**; **Figures 3A, C, E, G**). However, plant height was positively correlated with seed mass in other monocots and dicots, no significant correlation was found between these variables in palms and gymnosperms (**Table 2**; **Figures 3B, D, F, H**).

**Table 2:** Multivariate models (PGLMM) constructed with seed mass (fruit width) as response variable.

Taxon	AIC	Predictor variable	Estimate (SE)	Z	*P*
Palms	19.4	Blade length	0.127 (0.023)	5.539	< 0.001
		Stem height	0.023 (0.021)	1.102	0.271
Other monocots	574.9	Leaf size	0.140 (0.050)	2.831	0.005
		Plant height	0.242 (0.054)	4.487	< 0.001
Dicots	3743.0	Leaf size	0.162 (0.026)	6.283	< 0.001
		Plant height	0.390 (0.033)	11.935	< 0.001
Gymnosperms	105.8	Leaf size	0.345 (0.172)	1.998	0.046
		Plant height	0.056 (0.150)	0.371	0.711

**Figure 4:**
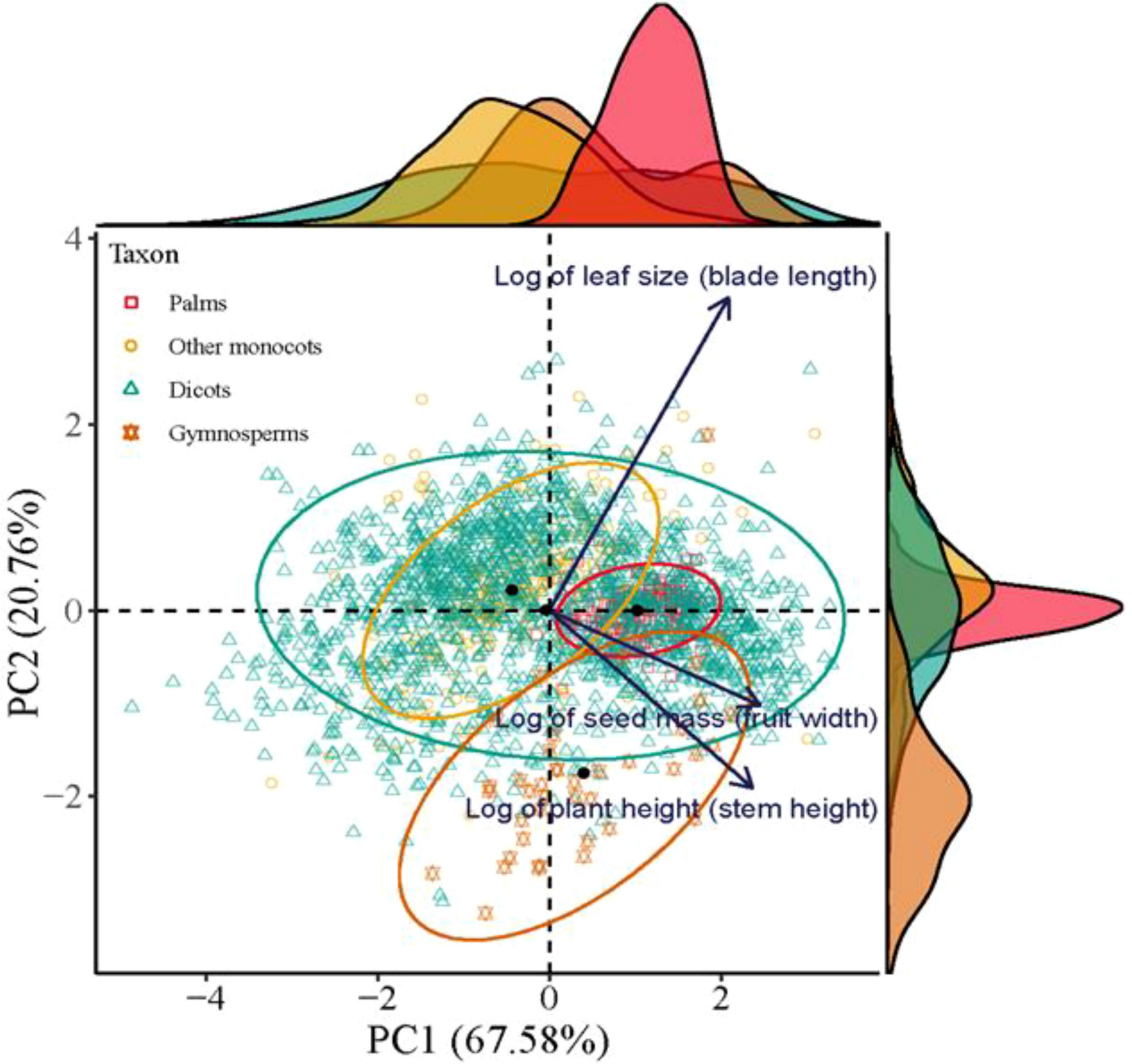
The location of palms, other monocots, dicots, and gymnosperms in a multivariate trait space illustrated by the first two axes of a principal component analysis (PCA) based on trait information on seed mass (fruit width), leaf size (blade length), and plant height (stem height). All data were log-transformed. Nonparametric multivariate analysis of variance (per-MANOVA) shows significant overall shifts in community structure (*P* < 0.001, R^2^ = 0.154, permutations = 999). PC1 and PC2 together account for 87.3% of the variability in the data.

The first two axes of the Principal Component Analysis (PCA) together accounted for 87.3% of variability in the functional traits of plant species (*p* < 0.001, R^2^ = 0.154, permutations = 999). The principal axis (PC1) was determined positively by seed mass and plant height. The second axis was significantly and positively correlated with leaf area (**Figure 4**). Thus, dicots were ordinated in a triangle of multivariate space, while palms, gymnosperms and other monocots were ordinated in three separated spaces, with large-seeded palms at the positive extreme of PC1 and small-leaved gymnosperms at the negative extreme of PC2 (**Figure 4**).

**Figure 5:**
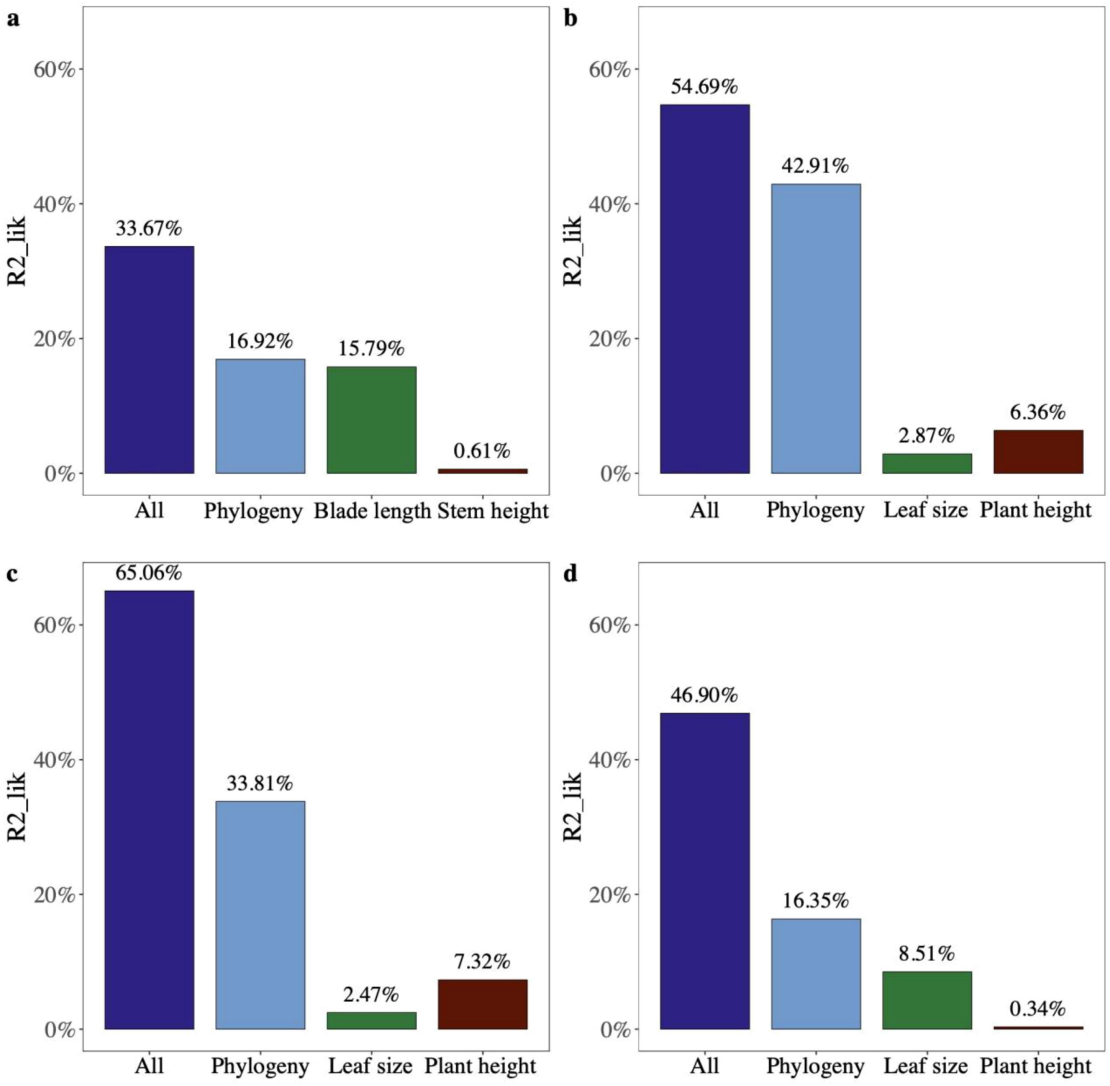
The relative contribution of different factors to the variation in seed mass (fruit width) of **(A)** palms, **(B)** other monocots, **(C)** dicots, and **(D)** gymnosperms using partial R^2^ for the logistic regression model.

The partial R^2^ for the logistic regression model showed that leaf size and phylogeny explained the vast majority of variation in seed mass across palm species (partial R^2^lik = 15.79%, ΔlogLik = 13.7, *p* < 0.001; R^2^lik = 16.92%, ΔlogLik = 14.8, *p* < 0.001; **Figure 5A**), while phylogeny and plant height explained variation in seed mass of species of other monocots and dicots (R^2^lik = 42.91%, ΔlogLik = 78.2, *p* < 0.001; R^2^lik = 6.36%, ΔlogLik = 9.2, *p* < 0.001; R^2^lik = 33.81%, ΔlogLik = 330.5, *p* < 0.001; R^2^lik = 7.32%, ΔlogLik = 60.9, *p* < 0.001; **Figures 5B, C**). Phylogeny rather than leaf size explained a majority of variation in seed mass in gymnosperms (R^2^lik = 16.35%, ΔlogLik = 3.6, *p* = 0.008; R^2^lik = 8.51%, ΔlogLik = 1.8, *p* = 0.059; **Figure 5D**).

## Discussion

In this study, we presented the first systematic quantification of major plant functional traits for palms, other monocots, dicots and gymnosperms and analyzed their relationships to leaf size, plant height and seed mass. Our findings reveal distinct patterns across plant clades, challenging the universal applicability of the Leaf-Height-Seed (LHS) strategy scheme.

Phylogenetic signal in functional traits of all plant clades distributed unimodally, except for stem height in the palm clade (Herben et al., 2018). Weak phylogenetic signal of stem height of palms indicates that phylogeny fails to constrain plant height across palm species, reflecting the uniqueness of functional traits of palms across the world (Sylvester and Avalos, 2013; Ma et al., 2015; Barrett et al., 2019). In contrast, the strong phylogenetic signal observed in plant height, leaf size and seed mass essentially reflects the four dominant phylogenetic groups that differ in major functional traits (Zheng et al., 2017; Collyer et al., 2021). This aligns with the growing consensus that strong phylogenetic signals across species result from trait similarities among closely related species (Aizen et al., 2015; Fuzessy et al., 2021).

Much progress has been made in recent years outlining the LHS strategy scheme at species level (Westoby and Wright, 2006; Klimešová et al., 2015), but relatively little attention has been given to functional trait patterns across plant clades, especially at genus level. Although trait relationships will become weak when species groupings are merged (Falster and Westoby, 2005), our results provide evidence that seed mass, even at genus level, is positively and closely correlated to plant height and leaf area across plant species within the clade of dicots and other monocots. This suggests covariation between functional traits among plant species in these clades (Moles et al., 2005a; Moles et al., 2005b; Falster et al., 2018), supporting the notion that the LHS scheme may not fully describe the major variation of plant traits in dicots and other monocots.

Although broad leaves have evolved in gymnosperms, we failed to detect any correlation between seed mass, leaf area and plant height among the clade gymnosperms, implying that the LHS scheme appears to hold for understanding the trait spectra of gymnosperms (Cornelissen, 1999). However, palms appear to be unique phylogenetic group because leaf size rather than plant height is consistently and positively correlated with seed mass (Göldel et al., 2015), suggesting that leaf size is a key driver of variation of seed mass in the palm clade (Wright et al., 2004). Our partial R^2^ for the logistic regression model provided further evidence that leaf size (i.e., blade length) rather than stem height explained a majority of variation in seed mass across palm species, while plant height contributed more to variation in seed mass than leaf size across species within dicots and other monocots.

These distinctions between plant clades that adhere to the LHS strategy (gymnosperms) and those that deviate from it (palms, dicots, and other monocots) provide valuable insights into the diverse evolutionary strategies employed by different plant groups. The adherence of gymnosperms to the LHS scheme may reflect their more conserved evolutionary history and relatively stable ecological niches. In contrast, the unique patterns observed in palms, dicots, and other monocots suggest more diverse adaptive strategies, possibly driven by their occupation of a wider range of ecological niches and their more recent evolutionary diversification.

Moreover, the PCA ordinated identified palms, other monocots, dicots, and gymnosperms in the respective location in a multivariate trait space. Trait correlations within each clade are considered to be caused by divergent patterns of correlated evolution of traits that inherited by its descendant lineages (Westoby et al., 2002; Messier et al., 2010).

Seed mass is an important ecological character affecting many aspects of plant ecology (Moles et al., 2005), because its variation can span 10 orders of magnitude across plant species (Rees and Venable, 2007). It is generally believed that seed mass has been the representative of dispersal ability, competitiveness and survival (Zhang et al., 2020), imposing great impacts on plant regeneration strategies and diversity of community. We showed that plant height scales positively with seed mass both in the clades of dicots and other monocots, which is consistent with previous meta-analyses of functional traits showing that plant size and seed mass are positively correlated (Moles et al., 2005a; Pierce et al., 2014; Díaz et al., 2015). However, a positive relationship between leaf size and seed mass observed in dicots and other monocots is contrary to previous studies demonstrating that seed size does not scale consistently with leaf size (Cornelissen, 1999; Wright et al., 2007), possibly due to our analysis at the genus level and the inclusion of a large dataset of species (Ke et al., 2022).

By comparing palms with other monocots, however we found that leaf size rather than plant height appears to be a consistent function of variation in seed mass (Winkel et al., 2001; Santini et al., 2017). As the main organ of photosynthesis in plants (Price et al., 2014), the limited numbers of large leaves of palms contribute a lot to seed development (Givnish, 1987; Onstein et al., 2017). The correlation of leaf size with seed mass may reflect strong natural selection for shade tolerance in understory palms (Göldel et al., 2015; Ma et al., 2015). In our study, there is a lack of close correlation of seed mass with plant height and leaf size in the extant gymnosperms, implying that variation in seed size of gymnosperms is mainly structured by dispersal syndrome and cone morphology (Leslie et al., 2017). Another possible explanation can be that extant gymnosperms exhibit a narrow range of seed sizes and lack very small seeds (Moles et al., 2005b).

In conclusion, our meta-analysis provides strong support to our prediction that the LHS strategy scheme does not consistently identify plant functional trait patterns across plant clades. However, LHS scheme captures a substantial part of the same spectra of strategy variation within each plant clade. The findings of our study provide important insights into better understanding seed mass correlations with plant height and leaf size across plant clades. Our study significantly contributes to the body of knowledge on the evolution and variation of plant functional traits, which are crucial in shaping plant life history strategies. By revealing clade-specific patterns, we highlight the complex interplay between evolutionary history and ecological adaptations. This understanding is fundamental in predicting plant responses to environmental changes and informing conservation strategies. Future research should focus on exploring the evolutionary and ecological drivers behind these clade-specific patterns, as well as their implications for plant community assembly and ecosystem functioning in a changing world.

## Data Availability

The raw data supporting the conclusions of this article will be made available by the authors, without undue reservation.
