# Supplementary material for: Palms are unique: clade‐level pattern of the leaf-height-seed strategy scheme
Source: Front Plant Sci. 2024 Nov 1;15:1465935. doi: 10.3389/fpls.2024.1465935 (PMC11563973; doi:10.3389/fpls.2024.1465935)
Supplement: Supplementary file 1 [file Table1.docx]

FIGURE S1
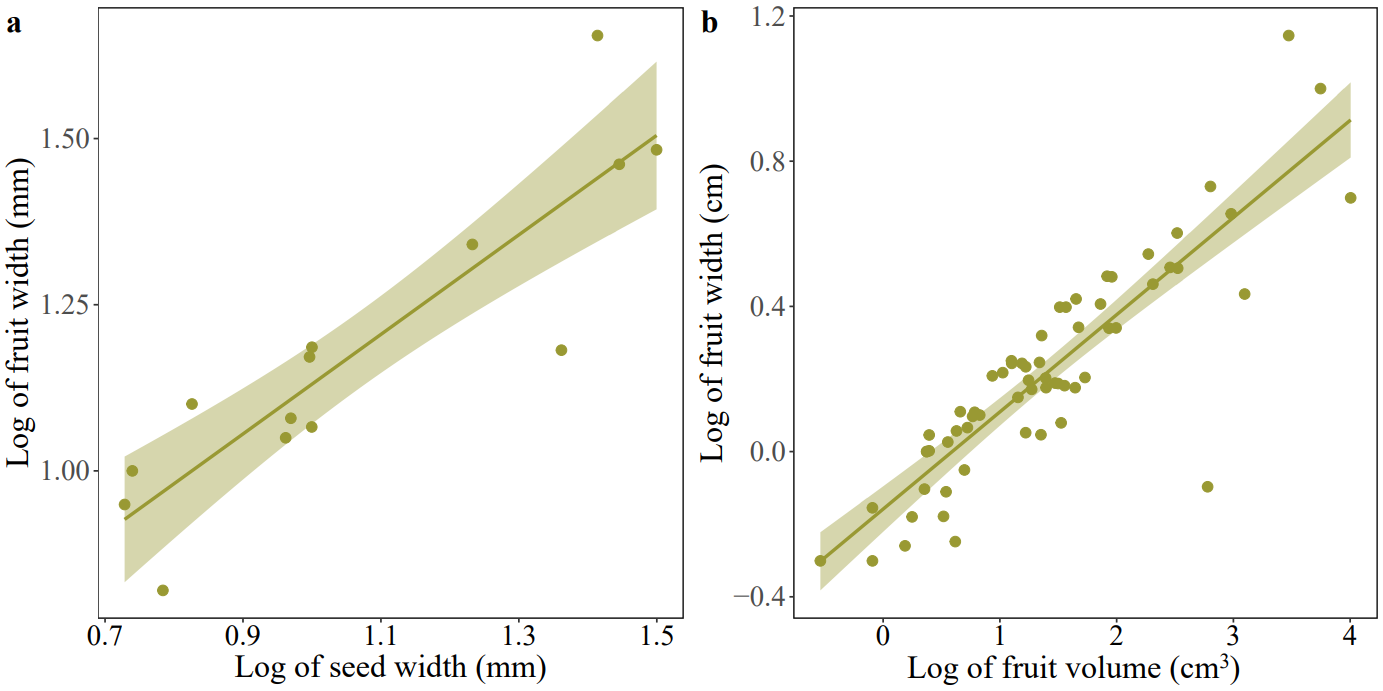


**FIGURE S1 Correlation of fruit width with seed width (a) and fruit volume (b) of palms. Log transformation was performed on the data.**
